# Synthesis of zeolitic imidazolate framework-8 and gold nanoparticles in a sustained out-of-equilibrium state

**DOI:** 10.1038/s41598-021-03942-0

**Published:** 2022-01-07

**Authors:** Brigitta Dúzs, Gábor Holló, Gábor Schuszter, Dezső Horváth, Ágota Tóth, István Szalai, István Lagzi

**Affiliations:** 1 Laboratory of Nonlinear Chemical Dynamics, Institute of Chemistry, Eötvös Loránd University, 1117 Pázmány Péter sétány 1/A, Budapest, Hungary; 2 Department of Physics, Budapest University of Technology and Economics, 1111 Budafoki út 8, Budapest, Hungary; 3 MTA-BME Condensed Matter Physics Research Group, Budapest University of Technology and Economics, 1111 Budafoki út 8, Budapest, Hungary; 4 Department of Physical Chemistry and Materials Science, University of Szeged, 6720 Rerrich Béla tér 1, Szeged, Hungary; 5 Department of Applied and Environmental Chemistry, University of Szeged, 6720 Rerrich Béla tér 1, Szeged, Hungary

**Keywords:** Chemistry, Materials science

## Abstract

The design and synthesis of crystalline materials are challenging due to the proper control over the size and polydispersity of the samples, which determine their physical and chemical properties and thus applicability. Metal − organic frameworks (MOFs) are promising materials in many applications due to their unique structure. MOFs have been predominantly synthesized by bulk methods, where the concentration of the reagents gradually decreased, which affected the further nucleation and crystal growth. Here we show an out-of-equilibrium method for the generation of zeolitic imidazolate framework-8 (ZIF-8) crystals, where the non-equilibrium crystal growth is maintained by a continuous two-side feed of the reagents in a hydrogel matrix. The size and the polydispersity of the crystals are controlled by the fixed and antagonistic constant mass fluxes of the reagents and by the reaction time. We also present that our approach can be extended to synthesize gold nanoparticles in a redox process.

## Introduction

The main aim of chemistry is to design and synthesize materials with new and unique properties that can be used in various types of applications (e.g., catalysis, drugs, energy production, and storage). One of the simplest and most widely applied techniques for the production of materials ranging from laboratory to industrial scales is the bulk synthesis method, in which the reagents are homogeneously distributed, and their concentrations gradually decrease due to the chemical reactions until the system reaches its thermodynamic equilibrium. In most cases, the chemical and physical properties of the materials (such as the average size, dispersity, and crystalline structure) depend on the growth rate of the products^1,2^. This process is especially crucial in the synthesis of crystalline materials, in which the local mass fluxes of the chemical species near the crystal particles determine the morphology, average size, and polydispersity of the samples.

In recent years, we have seen a growing interest in exploring new synthetic chemistry directions by using flow and non-equilibrium techniques. Notably, several promising attempts have been made to apply flow chemistry in the pharmaceutical industry to minimize waste and energy consumption and implement continuous drug quality control^2^. The main advantage of these techniques is the kinetic control over the synthetic process that allows the production of materials with unique and desired properties, especially in supramolecular chemistry^3,4^. Among others, a challenging direction in non-equilibrium synthesis is to apply control over both space and time, i.e., in which concentration gradients dictate the material properties in space^5^.

Metal − organic frameworks (MOFs) are unique materials consisting of metal ions and organic linkers, having one-, two-, or three-dimensional structures by the coordination of the ions and linkers^6–9^. MOFs have successfully been employed in gas storage^10–13^, separation^14^, heterogeneous catalysis^15^, targeted drug delivery^16^, and electronics^17^, and their hybrid materials, e.g., the multifunctional membranes are potential candidates in gas and liquid purification, and may be applicable as catalysts as well^18^. The most robust and frequently selected technique for the generation of MOFs is the solvothermal method^19^. In this approach, the experimental conditions, such as the solvent type, temperature, initial concentrations of the reagents, are important in controlling the morphology and the final size of the MOF crystals. The vast majority of the synthesis methods fall into the bulk and flow chemistry categories. The latter one has an inevitable advantage in upscaling and optimization of synthesis methods^20–25^. However, in both cases, the concentrations of the reagents decrease in the homogeneous reactor (bulk method) and fluid parcel in the flow reactor (flow chemistry method), which involves a decrease of the local mass fluxes of the reagents as well. This continuous decrease affects the growth mechanism of the crystalline particles thus limiting the final size of the crystals produced.

In the past few years, a new approach has been introduced for the production of MOFs in which the mass fluxes of the reagents are controlled through diffusion either in crosslinked hydrogels or in microfluidic networks. With precise control over the nucleation and growth of MOF crystals utilizing the diffusion processes, it is possible to achieve the formation of single crystals of peptide-based MOFs^26^, shaping MOF crystals^27^, and control the size of the MOF particles in a solid agarose hydrogel^28,29^. In these setups, one of the components is placed in the gel and the other one diffused from a reservoir, and at a given location in the gel, the formation of MOFs is governed by the time-dependent diffusion fluxes of the reagents. One attempt has been made to overcome the time-dependent mass fluxes, namely, the application of microfluidic networks, which provide a continuous supply of the reagents^26^. However, in this setup, the large-scale production of MOFs remains challenging and an open problem.

Crystallization in gelled media is a well-known method for generating single crystals and periodic precipitation because the gel matrix prevents the sedimentation of the produced crystal particles and the hydrodynamic instabilities, thus facilitating the crystal growth^30–33^. Studies on the synthesis of various MOFs have focused rather on the size control, and less is known about how the size and polydispersity of the crystals are linked together. In some applications, the polydispersity of the product plays a crucial role; for instance, the toxicity and catalytic activity of metal nanoparticles highly depend on polydispersity^34–39^. Therefore, the control of both the size and polydispersity of the crystals is one of the most challenging tasks, especially in non-bulk synthesis methods. An additional challenge is that usually bigger crystals (with the size of greater than a few micrometers) of MOFs can be achieved only using non-aqueous solvents at elevated temperature (*T* > 100 °C)^40,41^.

**Figure 1:**
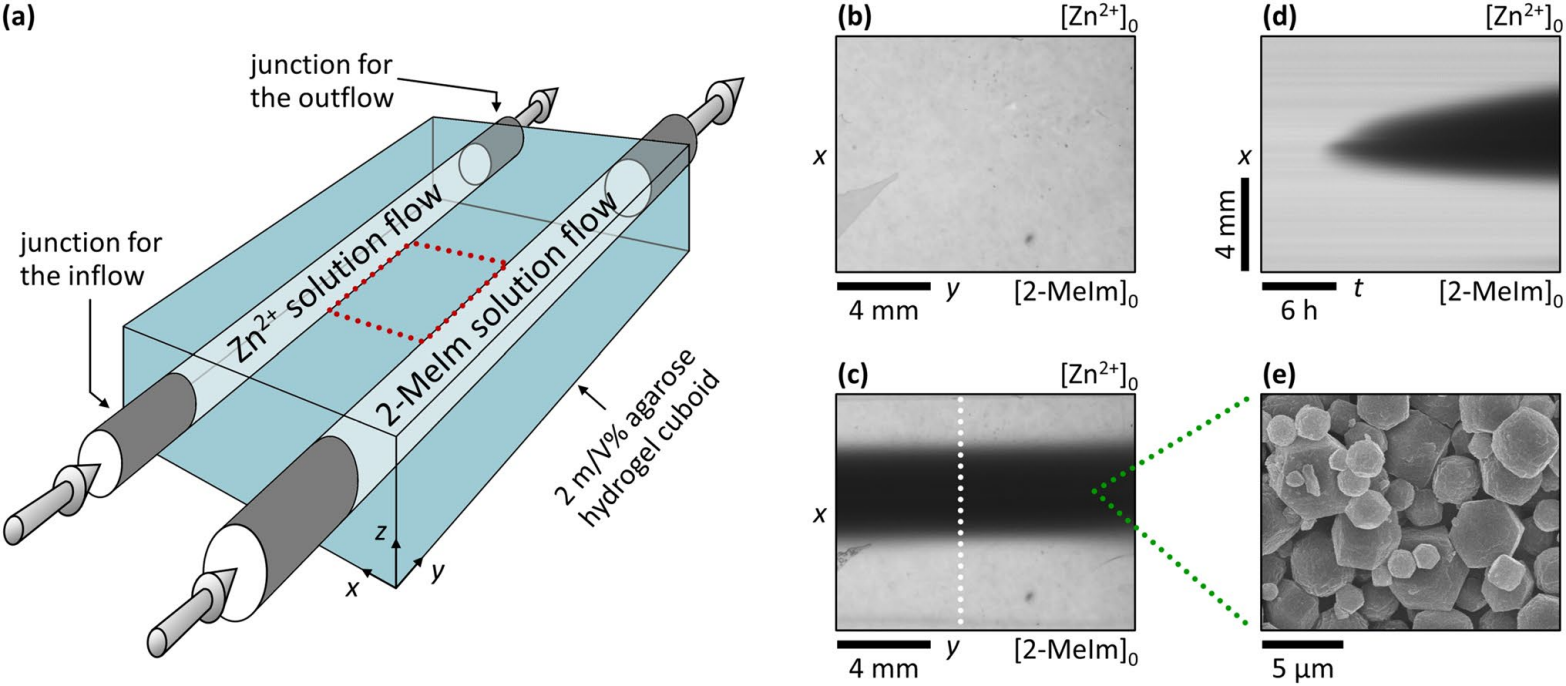
Synthesis of ZIF-8 crystals in a reaction–diffusion process at fixed antagonistic concentration gradients. Scheme of the gel reactor used in the synthesis (**a**), where 3D arrows represent the continuous flows of the fresh reagent solutions in the channels. Top view of the inter-channel zone (indicated by red dotted frame in (**a**)) at *t* = 0 (**b**) and 24 h (**c**). Space–time plot of the evolution of the precipitation zone (**d**) obtained along the white dotted lines shown in (**c**). The white precipitate appears as black in the pictures because transmitted light was monitored. The labels [Zn^2+^]_0_ and [2-MeIm]_0_ indicate the positions of the channels. A representative SEM micrograph of the particles isolated from the middle of the precipitate zone (**e**). The applied boundary concentrations were [Zn^2+^]_0_ = 11.25 mM and [2-MeIm]_0_ = 112.5 mM, respectively.

To address this drawback and the challenge of the size and polydispersity control, we present a non-equilibrium synthesis method for the generation of zeolitic imidazolate framework-8 (ZIF-8) utilizing the maintained and fixed cross-gradients of the reagents in time (zinc nitrate and 2-Methylimidazole (2-MeIm)) in a solid hydrogel at room temperature, using a two-channel open gel reactor (Fig. 1a)^42^. The formation of ZIF-8 is chosen because it has been considered a model reaction and is one of the most studied and understood reactions in the synthesis of MOFs. The synthesis is completed in *N*,*N*-dimethylformamide (DMF) and water mixture at a ratio of 1:1 at 25.0 ± 0.1 °C, typically within 1–2 days; the experimental details are given in Materials and Methods, Supplementary Information and Supplementary Figs. S1 and S2.

## Results and discussion

In our experimental setup, the solid agarose gel matrix was reagent-free (‘empty’) at the beginning of the synthesis (Fig. 1b), and then it was continuously fed by diffusion from the two embedded reagent flows having constant concentrations. This provided fixed boundary concentrations at the channel/gel interface, and, after some time, constant gradient fluxes were generated and maintained in time, and the reaction took place at the contact zone of two counter-propagating diffusive fronts creating a localized crystallization zone (Fig. 1c). The distance of the channels, i.e., the spatial extent of the inter-channel zone in direction *x*, was *w* = 10 mm. The ratio of concentrations of the reagents was varied to investigate its effect on the appearance of the crystallization zone in the gel while keeping the precipitation zone in the middle of the inter-channel gel zone (at *x* ~ 5 mm). We found the optimal ratio as [Zn^2+^]_0_: [2-MeIm]_0_ = 1:10, where the crystallization zone widened almost symmetrically in a diffusion-controlled way (Fig. 1d) until it reached a constant thickness in the course of time. Interestingly, moving precipitation fronts and the periodic precipitation of the ZIF-8 were observed if the initial concentrations were higher and the ratio of them was [Zn^2+^]_0_: [2-MeIm]_0_ = 1:1 (see Supplementary Fig. S3). The produced crystallization zone widened towards the channel having the 2-MeIm solution, highlighting that the optimal formation of ZIF-8 required an excess of the linker^43–45^. The phase purity and the structural identity of the synthesized ZIF-8 crystals were determined by using powder X-ray diffraction (PXRD) measurement. The recorded patterns exhibit sharp peaks that match the reported ones in the literature (see Supplementary Fig. S4).

Due to the porous structure of the gel medium, the convection was eliminated, and due to the fast nucleation of ZIF-8 particles^46^, its formation was much faster in the middle part of the agarose block than the rate of diffusion that delivered the fresh reactants there. Thus, the diffusion-limited nucleation and crystal growth and the macroscopic (Fig. 1c) and microscopic (Fig. 1e) characteristics of the ZIF zone were determined by the two antagonistic concentration gradients (i.e., how steep are the concentration gradients), which can be easily tuned by varying the channel concentrations.

### Concentration dependence in the out-of-equilibrium synthesis

**Figure 2:**
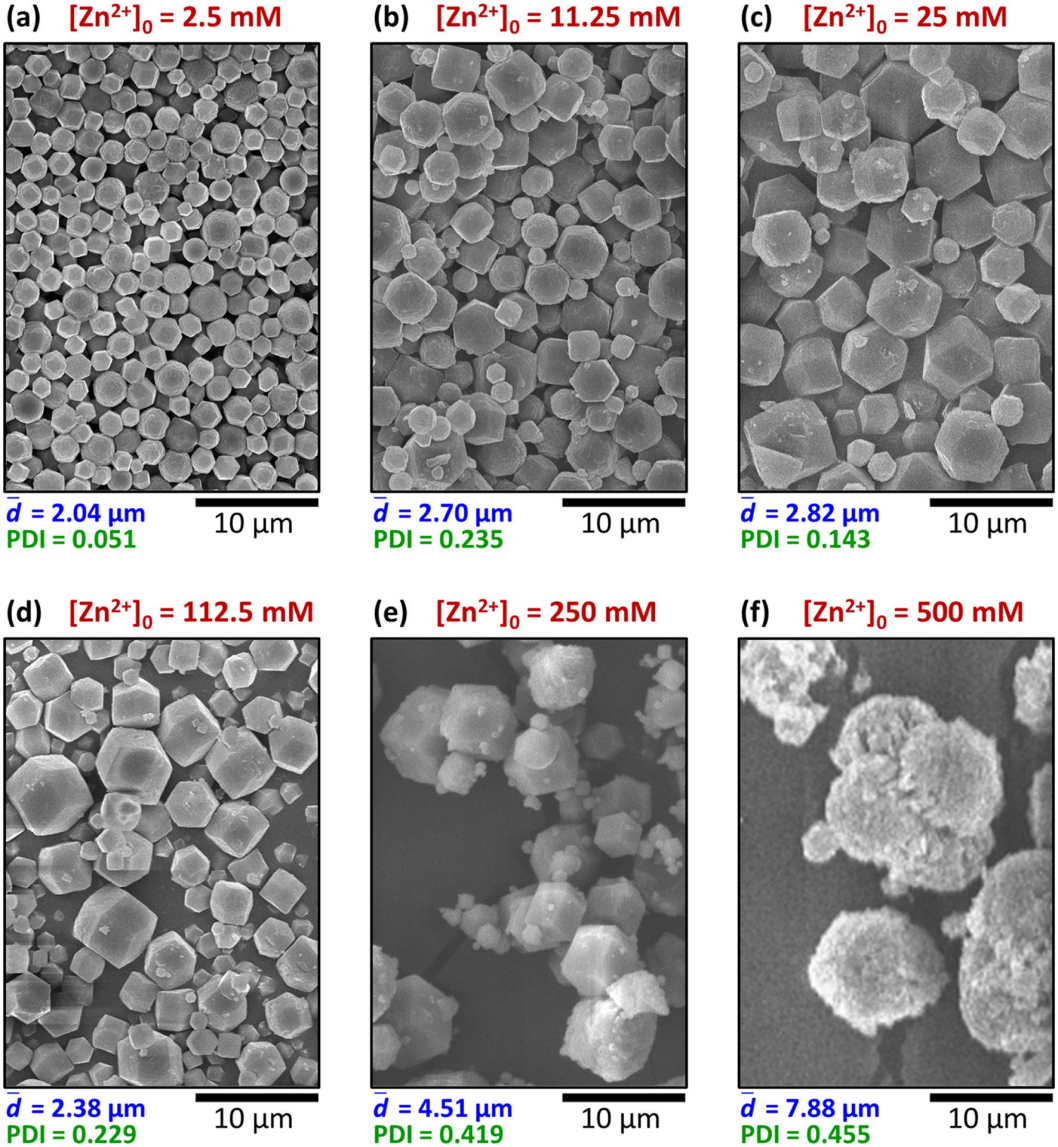
Concentration dependence of the particle size in the middle of the precipitate zone in the reaction–diffusion synthesis represented in SEM images (**a**–**f**). The ratio of the boundary concentrations was fixed to [Zn^2+^]_0_:[2-MeIm]_0_ = 1:10, and the synthesis time was 24 h. The average particle size $\left( {{\overline{d}}} \right)$ and the polydispersity index (PDI) are given below the SEM images.

**Figure 3:**
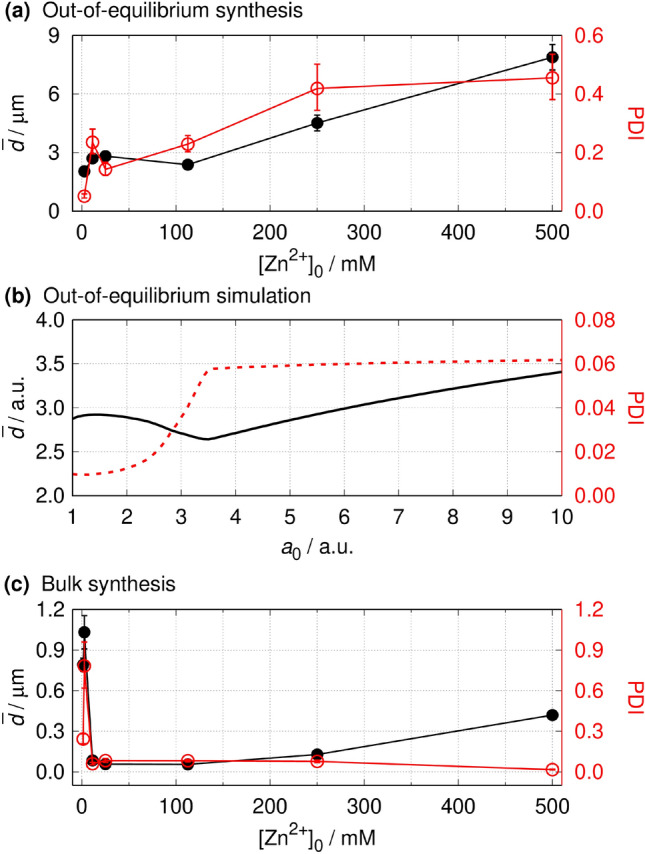
Concentration dependence of the average size and polydispersity in the middle of the precipitate zone in the reaction–diffusion synthesis in experiments (**a**) and simulations (**b**), and in case of the equilibrium bulk synthesis with fast mixing (**c**). The experimental conditions are the same as in Fig. 2. The error bars represent the 95% confidence interval of the measured data. The total length of the simulation was *t*_synt_ = 1.

We chose the synthesis time of 24 h and carried out a set of experiments between 2.5 and 500 mM of [Zn^2+^]_0_ using always a ten-fold excess of the linker (Figs. 2a–f). At lower boundary concentrations, the crystallization band appeared later and remained thinner, and the total amount of precipitate was less, as can be seen in snapshots recorded by a camera fixed above the setup (see Supplementary Fig. S5). We analyzed the morphology of the product by sampling the middle 1.5 mm-thick gel zone. Once the synthesis was finished, the zone of our interest was cut out, and the agarose matrix was removed by washing with DMF (for details, see Materials and Methods and Supplementary Fig. S1), and the sample was analyzed by scanning electron microscopy (SEM). The average of the particle diameters (${\overline{d}}$) and the polydispersity index (PDI) obtained from the SEM micrographs were used to characterize the samples. The details of the evaluation process can be seen in the SI. Below [Zn^2+^]_0_ = 2.5 mM, there was no detectable crystallization in the gel. At [Zn^2+^]_0_ = 2.5 mM, both the average size and polydispersity were relatively small (${\overline{d}}$ = 2.04 μm, PDI = 0.051), and typical dodecahedral shaped ZIF-8 particles formed (Fig. 2a). Following a slight increase up to [Zn^2+^]_0_ = 25 mM (Fig. 2c), the average size had a minimum at 112.5 mM (Figs. 2d and 3a). Further increase in [Zn^2+^]_0_ up to 250 mM resulted in a significant increase of the average particle size, which was accompanied by the increase of the polydispersity (${\overline{d}}$ = 4.51 μm, PDI = 0.419), while the morphology of the crystals remained unchanged (Fig. 2e). At [Zn^2+^]_0_ = 500 mM, large aggregates formed with a grainy surface on dodecahedron-like particles (Fig. 2f).

To gain more insight into the dynamics of the formation of MOFs and understand the unusual variation of the average size of MOF particles as a function of [Zn^2+^]_0_, we developed a dimensionless reaction–diffusion (RD) model incorporating diffusion, nucleation, and crystal growth. The mechanism of the MOFs formation contains *n* = 100 steps exhibiting successive growth of the crystal:

1 $${\text{A}} + {\text{B}} \to {\text{C}}_{{1}}$$

2 $$\begin{array}{*{20}c} {{\text{A}} + {\text{B}} + {\text{C}}_{{1}} \to {\text{C}}_{{2}} } \\ \cdots \\ \end{array}$$

3 $${\text{A}} + {\text{B}} + {\text{C}}_{{{\text{n}} - {1}}} \to {\text{C}}_{{\text{n}}} ,$$

where A and B denote the reagents, Zn^2+^ and 2-MeIm, respectively. For simplicity and transparency of the model, we consider a 1:1 ratio for both the stoichiometry and boundary concentration of the reagents. C_1_, C_2_, …, C_n-1_, and C_n_ are the crystals of increasing size. The reaction rate constants were set to *k*_1_ = 10 and *k*_2_ = *k*_3_ = … = *k*_n-1_ = *k*_n_ = 10^2^ to represent that the crystal growth is faster than the homogeneous nucleation (at the same supersaturation). Equation (1) represents the formation of the smallest crystals/nuclei, which is considered in the model as a concentration threshold-limited step, which is a usual treatment in models describing precipitation or crystallization^47^. The other steps (Eqs. (2) and (3)) represent the successive transformations of smaller particles into bigger ones (crystal growth), and they were similarly treated as concentration threshold-limited steps. The system can be described mathematically by the following set of partial differential equations in one dimension (in between and perpendicular to the parallel channels):

4 $$\frac{{\partial a}}{{\partial t}} = D_{{\text{A}}} \frac{{\partial ^{2} a}}{{\partial x^{2} }} - k_{1} ab\Theta \left( {ab - \alpha^{*}} \right) + r_{{\text{a}}}$$

5 $$\frac{{\partial b}}{{\partial t}} = D_{{\text{B}}} \frac{{\partial ^{2} b}}{{\partial x^{2} }} - k_{1} ab\Theta \left( {ab - \alpha^{*}} \right) + r_{{\text{b}}}$$

6 $$\frac{{\partial {c}_{{1}} }}{{\partial {t}}}={k}_{{1}} {{ab\Theta }}\left( {{{ab{-}\alpha^{*}}}} \right)+{ r}_{{1}}$$

7 $$\frac{{\partial c_{{\text{i}}} }}{{\partial t}} = r_{{\text{i}}} \;{\text{if}}\;i \in \left\{ {2, \ldots ,n} \right\},$$

where *a*, *b*, and *c*_i_ are the concentrations of A, B, and C_i_, and the system consists of *n* + 2 (i.e., 102) differential equations. *D*_A_ and *D*_B_ are the diffusion coefficients of the reagents in the gel. Θ is the Heaviside step function, and *r*_a_, *r*_b_, and *r*_i_ are the reaction terms describing the concentration change of A, B, and C_i_ due to the crystal growth, respectively. α* is the threshold concentration of the formation of C_1_ (nucleation; C_1_ is the smallest crystal in the model). For the crystal growth processes, we also considered threshold-limited reactions with a smaller threshold concentration than α*. The first terms on the right-hand side of Eqs. (4) and (5) describe the diffusion of the reagents in the gel. The second term in Eqs. (4) and (5), and also the first term in Eq. (6) is the rate of the concentration change due to the nucleation. In the model, we considered that crystals do not diffuse. The initial and boundary conditions were set to represent the experimental conditions, namely, *a*(*t* = 0, *x*) = *b*(*t* = 0, *x*) = *c*_i_(*t* = 0, *x*) = 0 (no chemical species in the gel at *t* = 0), and *a*(*t*, *x* = 0) = *b*(*t*, *x* = *L*) = *a*_0_, where *L* is the length of the domain. The details of the numerical model and the simulations can be found in the Supplementary Information.

The qualitative trends and tendencies in the average particle size (having a minimum) and the initial increase and then the stabilization of the polydispersity were reproduced in the simulations as well (Fig. 3b). When the boundary concentrations were smaller, the mass flux of the reagents was also small, causing small supersaturation in the middle zone. This resulted in the formation of few nuclei, and then the supply of the reagents was not efficient enough to exceed the nucleation product; thus, the growth of the existing nuclei was favored compared to the formation of new ones. This resulted in the formation of smaller but more monodisperse particles. At higher boundary concentrations, the mass flux of the reagents in the middle zone was sufficiently high to ensure not only the growth of the initially formed particles but also the formation of new ones. The appearance of small particles decreased the average size but increased the polydispersity dramatically (Fig. 3b, *a*_0_ = 3.4). Further increase of the boundary concentrations resulted in the increase of the average size because many nuclei formed at the beginning due to the high level of supersaturation. There was enough supply of the reagents to maintain the growth of the larger crystals without forming a significant number of new ones. The growth of the diameter of the large-surface crystals was slower than that of the small-surface ones, and the non-equilibrium conditions hindered the Ostwald ripening. These effects caused the conservation of the polydispersity at higher boundary concentrations. The results of the simulations supported these findings (see Supplementary Fig. S6). When the boundary concentration was small, the sample had well-defined Gaussian distribution with less polydispersity and fewer very small particles (see Supplementary Fig. S6a). However, when the boundary concentration was increased, the Gaussian distribution widened, smoothened, and became slightly asymmetric: particles with smaller sizes appeared in the sample, causing a slight decrease in the average size (see Supplementary Fig S6a, b and c). Further increase of the boundary concentration resulted in the appearance of bigger particles while the number of smaller particles does not change significantly (see Supplementary Fig. S6 d and e). Hence, the average size increased with increasing polydispersity (Fig. 3b).

To underline the advantage of the out-of-equilibrium synthesis performed at fixed antagonistic concentration gradients in the two-channel gel reactor, we carried out the bulk synthesis of MOFs using the same initial concentrations of the reagents (mixing and waiting for *t* = 24 h). Figure 3c and Supplementary Fig. S7 show that only submicron particles can be created in case of bulk synthesis, but with a smaller polydispersity than in the presented out-of-equilibrium process, and there is around one order of magnitude difference in the sizes of MOF crystals. The characteristic ZIF-8 crystal structure was observed only at the smallest concentration (see Supplementary Fig. S8).

### Time dependence in the out-of-equilibrium synthesis

**Figure 4:**
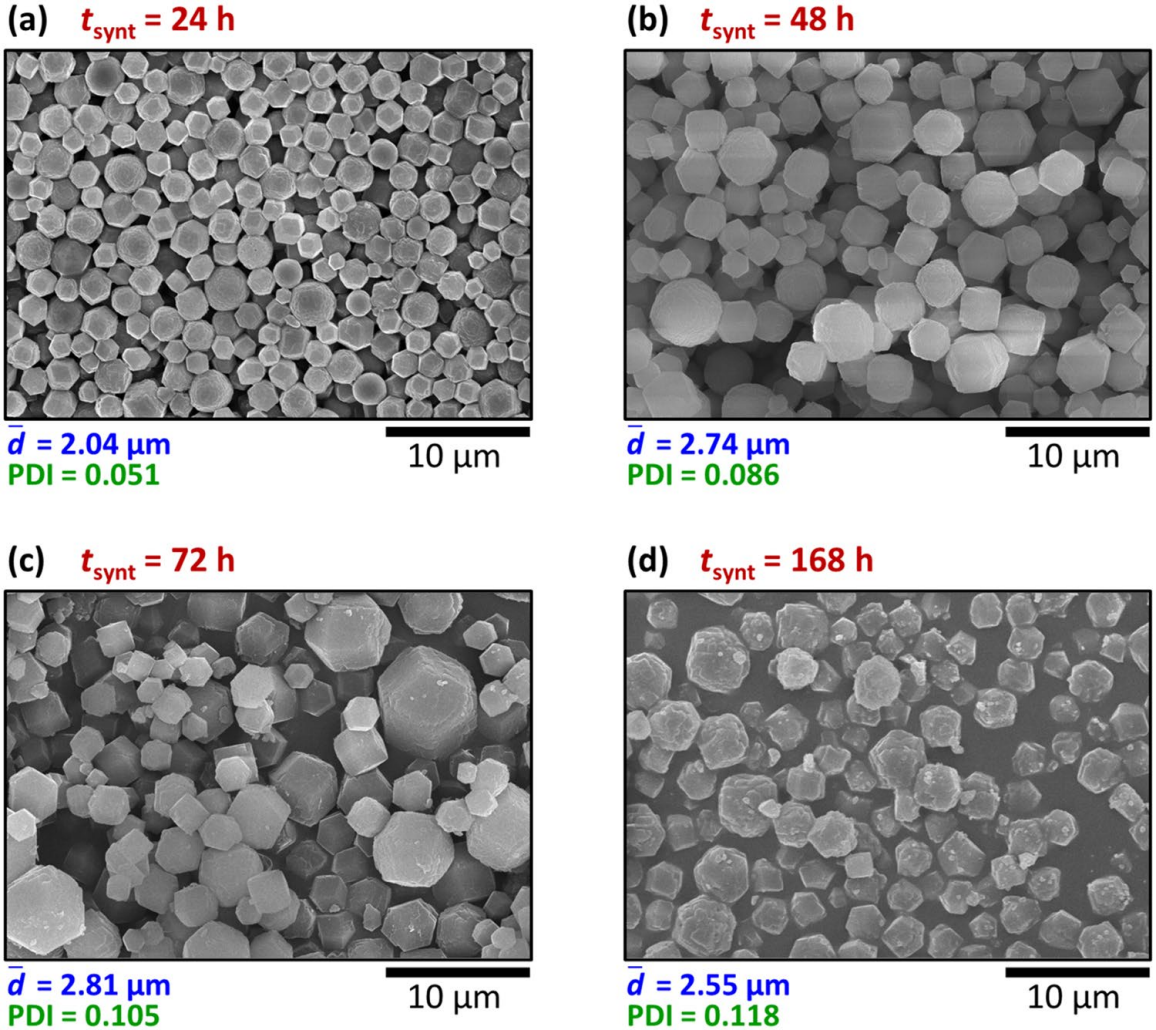
Synthesis time (*t*_synt_) dependence of the particle size in the middle of the precipitate zone in the reaction–diffusion synthesis represented in SEM images (**a**–**d**). The boundary concentrations were [Zn^2+^]_0_ = 2.5 mM and [2-MeIm]_0_ = 25 mM in every case. The average particle size $\left( {{\overline{d}}} \right)$ and the polydispersity index (PDI) are given below the SEM micrographs.

A key factor in the synthesis of materials is the synthesis time and how it affects the physical and chemical properties of the final product, such as the morphology, the average size, and the polydispersity. We synthesized MOFs (fixing the concentrations of the reagents at [Zn^2+^]_0_ = 2.5 mM and [2-MeIm]_0_ = 25 mM) extending the time from 24 to 168 h. We observed that the average size of the MOF crystals, having a dodecahedral shape, increased in time (Figs. 4a–c). The polydispersity also increased, but the change was less steep at higher synthesis times. Similar behavior was captured by the model as well (see Supplementary Fig. S9). When the synthesis time was increased at *a*_0_ = 5.0, the Gaussian distribution widened and shifted towards bigger particles resulting in an increase in the average size with increased polydispersity at the beginning. The average size increased further during the next period, but the polydispersity increased only slightly. However, in the experiments, the further extension of the synthesis time (to 168 h) resulted in a degradation of the particles, and the average size was smaller and the PDI was greater than after 72 h (Fig. 4d). This degradation of the formed crystals (less sharp crystal edges and indistinct facets) can be due to the dissolution of MOFs staying longer in the solvent, which suggests the use of 24–72 h synthesis time in the given experimental setup^48^.

### Spatial position dependence in the out-of-equilibrium synthesis

**Figure 5:**
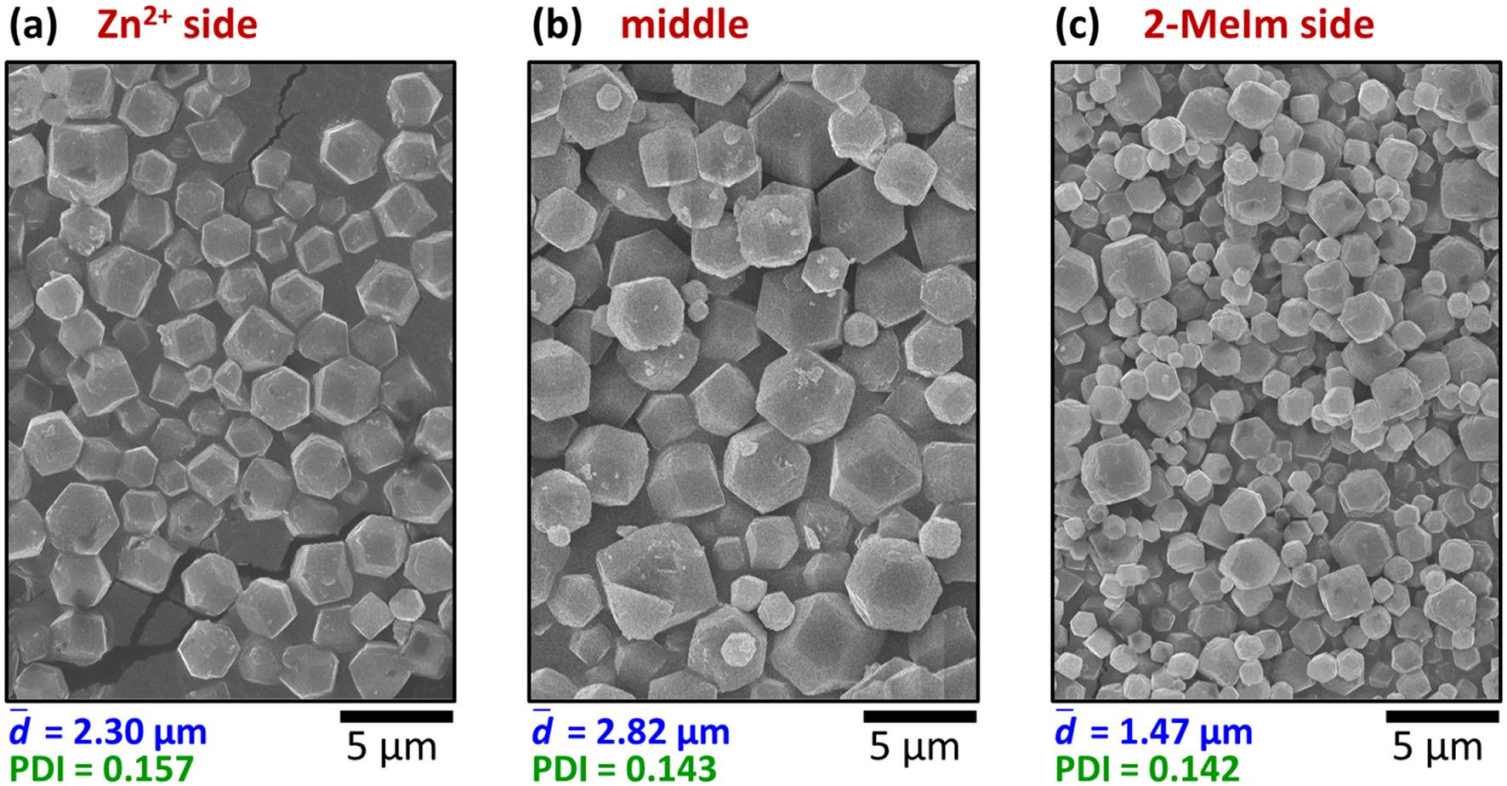
Particle size at different spatial positions along the direction of the cross-gradients (Zn^2+^ side, middle zone, and 2-MeIm side) in the out-of-equilibrium synthesis represented in SEM micrographs (**a**–**c**). The boundary concentrations were fixed to [Zn^2+^]_0_ = 25 mM and [2-MeIm]_0_ = 250 mM, and the synthesis time was 24 h. The average particle size $\left( {{\overline{d}}} \right)$ and the polydispersity index (PDI) are given below the SEM micrographs.

Finally, we wanted to explore the size distribution of the MOFs along a perpendicular line to the two channels since until now we only focused on the crystals formed at the middle part of the agarose block. We intended to investigate how the distance from the sources might affect the characteristics of the crystals. The inter-channel gel block was sliced parallel with the channels, and 1.5 mm-thick zones were analyzed from the middle of the crystallization zone and from the two sides (their positions are presented in Supplementary Fig. S1). The middle zone can be characterized by the largest particle size (Fig. 5b). Closer to the channels in both cases, smaller particles formed with almost the same polydispersity (Fig. 5a,b).

In general, a series of bulk experiments may suggest the optimal conditions of ZIF-8 crystal formation. However, in our setup, the optimal conditions were intrinsically determined by the antagonistic concentration gradients since these determined the spatial position (‘middle zone’), where the nucleation started. Since the middle zone was fed from two sides with the necessary reagent mass fluxes, the largest crystals developed here. At the same time, as the middle zone consumed the reagents, the local concentrations of Zn^2+^ and 2-MeIm along the respective sides were much lower, resulting in smaller crystals. The simulations, where there is no chemical difference between the metal ion and the linker, showed the same trend (see Supplementary Fig. S10), highlighting that our RD model with a successive crystal growth mechanism can qualitatively describe and reproduce all the basic features of the out-of-equilibrium synthesis of MOFs. The two sides were not symmetric in the experiments due to the [Zn^2+^]_0_: [2-MeIm]_0_ = 1:10 ratio and the 1:2 stoichiometry of the ZIF-8 formation^49–51^. As a result, the particles of the Zn^2+^ side were larger than that of the 2-MeIm side (compare Fig. 5a,c). Such a tendency was also observed in case of very long synthesis times (*t*_synt_ = 168 h) (compare ${\overline{d}}$ = 6.93 μm and ${\overline{d}}$ = 2.69 μm in Supplementary Figs. S11a and S11c, respectively), but in this case, the particle size in the middle zone was smaller (${\overline{d}}$ = 2.55 μm) due to the degradation of the product (see Supplementary Fig. S11b).

### Out-of-equilibrium synthesis of gold nanoparticles

**Figure 6:**
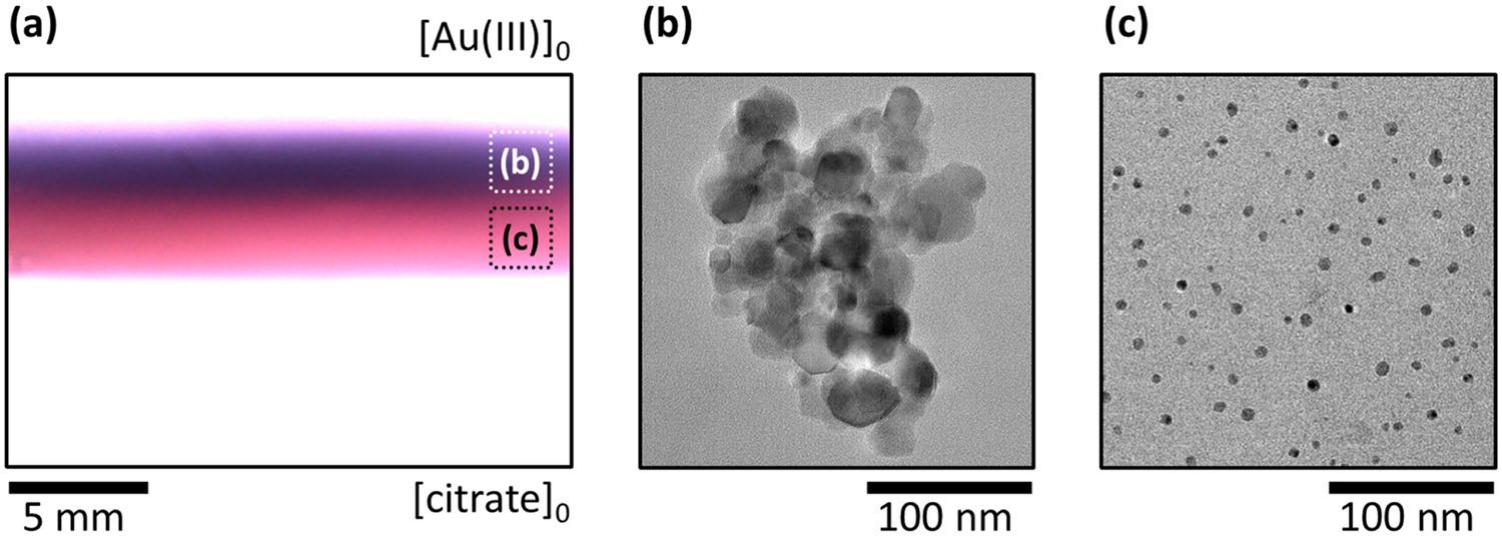
Synthesis of AuNPs in the two-channel gel reactor in the PAA gel. Top view of the inter-channel zone at the end of the experiment (*t* = 24 h) (**a**) and representative TEM micrographs of the particles isolated from the purple (**b**) and red (**c**) regions. The labels [Au(III)]_0_ and [citrate]_0_ indicate the positions of the channels. The applied boundary concentrations were [Au(III)]_0_ = 1 mM and [citrate]_0_ = 10 mM, respectively.

To illustrate the magnificent power of our approach and that it is not limited to the synthesis of MOFs, we have chosen another type of reaction, namely, a redox reaction, to produce gold nanoparticles (AuNPs). The citrate-based route in aqueous phase is one of the commonly used techniques, and the citrate not only reduces the Au(III) ions but also stabilizes the formed AuNPs via adsorption onto the crystal facets of the particles^52^. We carried out the synthesis in a similar manner; we used a four- or ten-fold excess of citrate ([Au(III)]_0_ = 1 mM), and the time of synthesis was reduced to 6–24 h (see Materials and Methods). Since the agarose (which is a polysaccharide) reduces Au(III) and facilitates the production of AuNPs^53^ (see Supplementary Fig. S12), we replaced the agarose hydrogel matrix with a polyacrylamide block. A purple zone appeared at 6 h, indicating the formation of AuNPs in the middle of the inter-channel gel domain. The product zone became wider and structured in 24 h (Fig. 6a). The purple zone contained larger nanoparticles (with a size between 10 and 50 nm) and their aggregates (Fig. 6b). The red zone consisted of smaller and non-aggregated AuNPs with ${\overline{d}}$ = 6.3 nm and PDI = 0.08 (Fig. 6c) due to the higher local excess of citrate. This observation is in good accordance with the fact that an increased concentration of citrate in bulk synthesis results in the decrease of the particle size since citrate is both the reducing and stabilizing/capping agent^52,54–56^. Interestingly, our method provided sub-10 nm particles at room temperature, which cannot be achieved in a classical citrate-based (Turkevich) method^57^ even at elevated temperature (~ 70–100 °C)^52,54,55^. To obtain sub-10 nm citrate-stabilized AuNPs, the usage of either a stronger reducing agent (e.g., sodium borohydride)^58^ or other additive (e.g., tannic acid)^59^ is needed.

We should emphasize two highly non-trivial aspects of our findings which present that the application of the out-of-equilibrium conditions in the synthesis of materials provides strikingly different characteristics of the samples compared to the bulk synthetic routes. Firstly, we observed the formation of ZIF-8 crystals close to the channel having Zn^2+^ ions (Fig. 5a) with almost the same characteristics as the crystals formed at the 2-MeIm side (Fig. 5c). This contradicts the experimental observations that only a high excess of 2-MeIm (higher than the stoichiometric ratio) favors the formation of ZIF-8, especially in the aqueous phase^43,45^. Secondly, we could generate AuNPs with sub-10 nm size using the classical citrate method. Based on these two observations showing the power of our method, we can conclude that although the nucleation is predominantly governed by the local concentrations of the reagents, the crystal growth can be effectively controlled by the mass fluxes of the reagents. In our experimental setup, constant mass fluxes are maintained, generating circumstances having the ability to synthesize materials either at conditions that cannot be realized in the bulk setup (i.e., the formation of the ZIF-8 in the excess Zn^2+^) or with better quality (i.e., the formation of AuNPs having smaller size with less polydispersity).

## Conclusions

In summary, we have demonstrated a general and facile method to synthesize ZIF-8 crystals and gold nanoparticles utilizing out-of-equilibrium conditions by using a continuous two-side-fed setup in a hydrogel matrix. In our approach, the continuous supply of the reagents is realized by diffusion in a gelled medium, which keeps the system out-of-equilibrium and affects the growth rate of the crystals. One of the most noticeable differences between the previously proposed synthetic methods based on diffusive fluxes of the reagents and our method is the presence of fixed concentration gradients due to the continuous feeding of the reagents. Our approach gives rise to the formation of significantly larger ZIF particles (ranging between one and two orders of magnitude) than a well-mixed batch process with the same initial concentrations of the reactants. The average size of ZIF-8 can be fine-tuned by easily controllable experimental parameters, such as the feed concentration, synthesis time, and the relative position in between the feed channels. The main advantages of the setup are that the spatial position of the synthesis and the growth rate of the crystals can be controlled by the ratio of the mass fluxes of the reagents and the magnitude of the gradient of the reagents, respectively. Another advantage is the scalability; it can be simply downscaled to microfluidic and upscaled to macrofluidic ranges, e.g., by increasing the *y*-direction reactor size we can multiply the product yield with the same particle properties. However, it should be noted that the polydispersity of the ZIF crystals cannot be independently controlled from the average size. The developed reaction–diffusion model incorporating the early stages of crystal growth describes qualitatively the experimentally observed phenomena. As we have shown, this out-of-equilibrium setup is not only limited to the synthesis of ZIF-8, it can also be successfully used to generate AuNPs as well.

We should make several important notes regarding our approach. Firstly, in this study, we used a continuous flow of the reagents in the channels to maintain fixed boundary concentrations, which generated more waste compared to the bulk synthesis methods. Our chief aim was to present a proof-of-concept of this non-equilibrium method. Secondly, from the engineering and environmental impact point of view, the synthesis can be carried out in a closed-loop continuous flow of the reagents in which the boundary concentrations can be slightly decreased. This decrease in the concentrations can depend on the volume of the solution in the closed-loop system, gel volume, and the amount of the product synthesized. Lastly, our setup can be easily adapted for the synthesis of various MOFs in the aqueous phase, which setup can decrease the environmental impact due to eliminating DMF in the synthesis^57,61^.

We believe that the presented approach can be used for the synthesis of other materials and be extended to synthesize composite structures and core–shell nanoparticles at the laboratory scale^62,63^. The synthesis can be performed not only in a hydrogel but also in other porous media, in which the mass transport can be realized by the combination of diffusion and advection or solely by advection.

## Materials and methods

### Metal-organic frameworks

#### Synthesis in the reaction–diffusion reactor

The synthesis was performed in a cuboid-shaped gel reactor with two flow-through channels. The gel body was made of 2 m/V% agarose (Sigma-Aldrich, A0169) with the dimensions of *l*_x_ = 30 mm, *l*_y_ = 40 mm, *l*_z_ = 10 mm. The distance between the closest points of the two parallel, cylindrical channels was *w* = 10 mm, the diameter of the channels was 4.5 mm. The thickness of the gel layers below and above the channels was *h* = 2.0 mm. The gel was prepared as follows: the agarose was dissolved in a 1:1 solution of DMF (VWR, ≥ 99.0%) and H_2_O in a 100 °C water bath. After complete dissolution, the melted gel was poured around two fixed plastic tubes in a Plexi mold and was covered by a Plexi plate. Once the gel had cooled and solidified, the mold tubes were pulled out, forming the two fluidic channels, and the junction tubes for the in- and outflows of the reagents were connected. A video of the gel preparation and the reactor is available as a supporting material of our previous publication^42^. The continuous flows of the reactant solutions in the channels were maintained by peristaltic pumps with a 100 mL/h flow rate. The reactants were separated as follows: channel A was fed with Zn(NO_3_)_2_·6H_2_O (Sigma-Aldrich, purum p.a., ≥ 99.0%) dissolved in DMF: H_2_O (1:1), channel B was fed with 2-MeIm (Sigma-Aldrich, 99%) dissolved in DMF: H_2_O (1:1). The chemicals were used without further purification, and the solutions were prepared daily with ion-exchanged water. The input feed concentrations of the reagents in the channels are indicated by [ ]_0_ in the text. The synthesis time was typically 24–72 h (indicated in the text). The reactor was thermostated to 25.0 ± 0.1 °C. The setup was enlightened by a white LED backlight (Advanced Illumination), and the pictures were recorded by a digital camera (Imaging Source DMK 330UX250) from the other side. For image processing, we used the ImageJ software.

#### Isolation of the product from the agarose matrix

The gel cuboid was removed from the mold and was sliced parallel with the channels to obtain samples of different zones along the cross-gradients. The thickness of each slice was 1.5 mm in direction *x*, and the position of them is indicated in Supplementary Figure S1. The slices were placed in 1.5–1.5 mL DMF to dissolve the agarose matrix during mild stirring (it typically took 5–8 min). Then the resulting dispersion was centrifuged (20 min, 30,000 RCF, 25.0 ± 0.1 °C), and the upper liquid (dissolved agarose and DMF) was removed by pipette. The washing of the remaining white solid powder was repeated twice; if needed, the redispergation was enhanced by sonication (10 s). After the final removal of DMF, the samples were desiccated at room temperature (typically for 24 h), and then the samples were stored in closed Eppendorf tubes until the SEM measurement.

#### Powder X-ray diffraction (PXRD) and scanning electron microscopy (SEM) measurements

Powder X-ray diffraction (PXRD) was applied to determine the crystalline phase of MOFs yielded in the RD system. Reactants were used in [Zn^2+^]_0_ = 25 mM and [2-MeIm]0 = 250 mM concentrations, and the synthesis time was set to 24 h. To collect the appropriate amount of solid sample, 9 parallel runs were performed, and the products were unified after isolation from the gel ingredients in a way introduced in the previous subsection. The dry precipitate sample was investigated with an X-ray diffractometer (Rigaku MiniFlex II Desktop X-ray Diffractometer) with CuKα (= 0.1542 nm) as radiation source at ambient temperature in the 5–60° 2Θ-range applying 0.02° step size. The recorded PXRD pattern was compared with literature references^43,64,65^. The perfect match of the recorded and published data proves that the precipitate particles produced in our RD system are ZIF-8 crystals. Besides the PXRD measurements, scanning electron microscopy (SEM, Hitachi S-4700, 10 kV accelerating voltage, gold sputtering) was also applied on the ZIFs to facilitate microstructure and size distribution analysis.

#### Bulk synthesis of metal − organic frameworks

Equal volumes of the initial solutions ([Zn^2+^]_0_ and [2-MeIm]_0_ concentrations are given in the text, and the solvent was DMF: H_2_O (1:1)) were mixed rapidly in a beaker. The mixing was carried out by using two manually released automatic pipettes: we added equal volumes (typically 2–2 mL) of the two reactant solutions to a beaker simultaneously, with one fast move, while the beaker was continuously stirred with a magnetic stirrer. The mixture was stirred for 2 min and then was stored at 25.0 ± 0.1 °C for the indicated synthesis time. After that, the dispersion was centrifuged, the DMF: H_2_O (1:1) solvent was removed, and the product was washed with 100% DMF two times (similarly as described in case of the out-of-equilibrium synthesis).

### Gold nanoparticles

#### Synthesis in the reaction–diffusion reactor

Two gel materials were tested, the 2 m/V% agarose and the polyacrylamide (PAA). We used the mold described above for the gel preparation in both cases. The agarose gel was prepared as in case of MOFs, but the solvent was ion-exchanged water. The PAA gel was prepared as follows: acrylamide (AA, Fluka, ≥ 99%) and *N*,*N*’-methylene-bisacrylamide (BAA, Sigma, ≥ 98%) were dissolved in 4.8 mL ion-exchanged water. Then 0.6 mL (NH_4_)_2_S_2_O_8_ (APS, Sigma) and 0.6 mL triethanolamine (TEA, Sigma, ≥ 99%) solutions were added dropwise, vigorously mixing for 1–2 min. The final concentrations were [AA] = 2.8 M, [BAA] = 13 mM, [APS] = 13 mM, [TEA] = 30 mM. The mixture was poured into the Plexi mold and was covered with a Plexi plate for 24 h. Then the channel forming tubes were pulled out, the gel body was removed from the mold, and was washed with ion-exchanged water for 96 h. Due to the washing, the whole gel body swelled, and the distance between the channels became *w* = 14 mm. After that, junction tubes (diameter 7.0 mm) were inserted into the channels, the PAA gel was placed into a bigger mold, was poured around with 2 m/V% hot agarose, and was covered with a Plexi plate. The continuous flows of the reactant solutions in the channels were maintained by peristaltic pumps with a 100 mL/h flow rate. The reactants were separated as follows: channel A was fed with an aqueous solution of HAuCl_4_·3H_2_O (Sigma-Aldrich, ≥ 99.9%), channel B was fed with an aqueous solution of sodium citrate tribasic dihydrate (Sigma-Aldrich, ≥ 99.0%). The chemicals were used without further purification, and the solutions were prepared daily with ion-exchanged water. The input feed concentrations of the reagents in the channels are indicated by [ ]_0_. The synthesis time was 6 h (in case of agarose gel) and 24 h (in case of PAA gel). The reactor was thermostated to 25.0 ± 0.1 °C. The setup was enlightened by a white LED backlight (Advanced Illumination), and the pictures were recorded by a color digital camera (Imaging Source DFK 41BF02.H) from the other side. For image processing, we used the ImageJ software.

#### Transmission electron microscopy (TEM)

The size distribution of the gold nanoparticles yielded in the RD system was determined with the aid of transmission electron microscopy (TEM). The experiment was carried out with FEI TECNAI G2 20 X-Twin high-resolution transmission electron microscope applying with 200 kV acceleration voltage. Before the measurements, the samples were dispersed in distilled water and then dropped and dried on a carbon film-coated copper grid (200 mesh).

## Supplementary Information

Supplementary Information.
