# Supplementary material for: Synthesis of zeolitic imidazolate framework-8 and gold nanoparticles in a sustained out-of-equilibrium state
Source: Sci Rep. 2022 Jan 7;12:222. doi: 10.1038/s41598-021-03942-0 (PMC8741818; doi:10.1038/s41598-021-03942-0)
Supplement: Supplementary file 1 — Supplementary Information. [file 41598_2021_3942_MOESM1_ESM.pdf]

Supplementary Information

Synthesis of zeolitic imidazolate framework-8 and gold nanoparticles in a sustained out-of-equilibrium state

Brigitta Dúzs, Gábor Holló, Gábor Schuszter, Dezső Horváth, Ágota Tóth, István Szalai,* and István Lagzi*

Abstract: The design and synthesis of crystalline materials are challenging due to the proper control over the size and polydispersity of the samples, which determine their physical and chemical properties and thus applicability. Metal−organic frameworks (MOFs) are promising materials in many applications due to their unique structure. MOFs have been predominantly synthesized by bulk methods, where the concentration of the reagents gradually decreased, which affected the further nucleation and crystal growth. Here we show an out-of-equilibrium method for the generation of zeolitic imidazolate framework-8 (ZIF-8) crystals, where the non-equilibrium crystal growth is maintained by a continuous two-side feed of the reagents in a hydrogel matrix. The size and the polydispersity of the crystals are controlled by the fixed and antagonistic constant mass fluxes of the reagents and by the reaction time. We also present that our approach can be extended to synthesize gold nanoparticles in a redox process.

Table of Contents

1 Experimental Procedures 3
1.1 Sample preparation procedure 3
1.2 Evaluation of the SEM micrographs 3
2 Numerical model and simulations 5
2.1 Reaction-diffusion (out-of-equilibrium synthesis) of MOFs (partial differential equations) 5
2.2 Bulk synthesis of MOFs (ordinary differential equations) 5
3 Supplementary Figures 6
4 References 9

1 Experimental Procedures

1.1 Sample preparation procedure

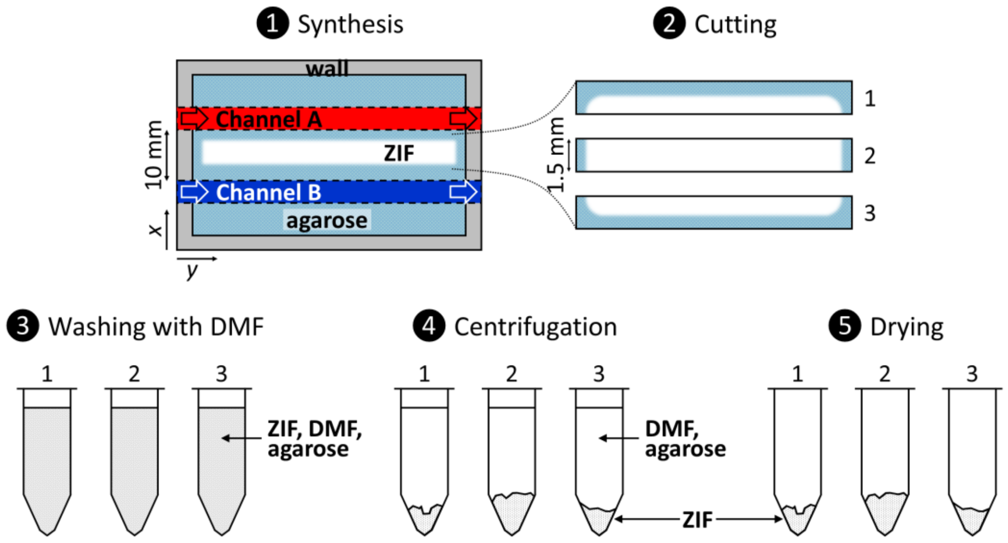

Supplementary Figure S1. Synthesis and sample preparation. After the synthesis (step 1, 24-168 h), we cut up the middle zone to 1.5 mm-thick slices (step 2, 1-2 min). The slices containing agarose and zeolitic imidazolate framework-8 (ZIF-8) crystals were washed with DMF to dissolve agarose (step 3, 5-8 min). The dispersion was centrifuged (step 4, 20 min), and the DMF was removed by a pipette (step 5, 1 min). Steps 3-5 were repeated twice, then the washing of the centrifuged sediment was enhanced by sonication (10 sec). Finally, the ZIF was let dry at room temperature for 24 h and then kept in closed Eppendorf tubes until the SEM measurement.

1.2 Evaluation of the SEM micrographs

We determined the diameters of all the visible particles in one image (150-350 particles): we approximated the particles with circles drawn and measured with the ImageJ software. The SEM micrographs and the corresponding histograms are shown in two selected cases: in Figures S2a and b, the particle size distribution is almost symmetric, and the polydispersity is smaller. In Figure S2c, the polydispersity is higher due to a significant number of large and ten times smaller particles in the same sample. This results in a size distribution skewed with a long right tail (Figure S2d). The average particle diameter ($\bar{d}$) and the polydispersity index (PDI) are calculated as follows:[1,2]

$$\bar{d} = \frac{\sum_{i=1}^{N_{tot}} d_i}{N_{tot}} \qquad \text{(S1)}$$

$$\text{PDI} = \left(\frac{\sigma}{\bar{d}}\right)^2, \text{ where } \sigma = \sqrt{\frac{\sum_{i=1}^{N_{tot}} (d_i - \bar{d})^2}{N_{tot} - 1}}, \qquad \text{(S2)}$$

where d_i are the diameters of the particles, N_{tot} is the number of the particles, and σ is the standard deviation.

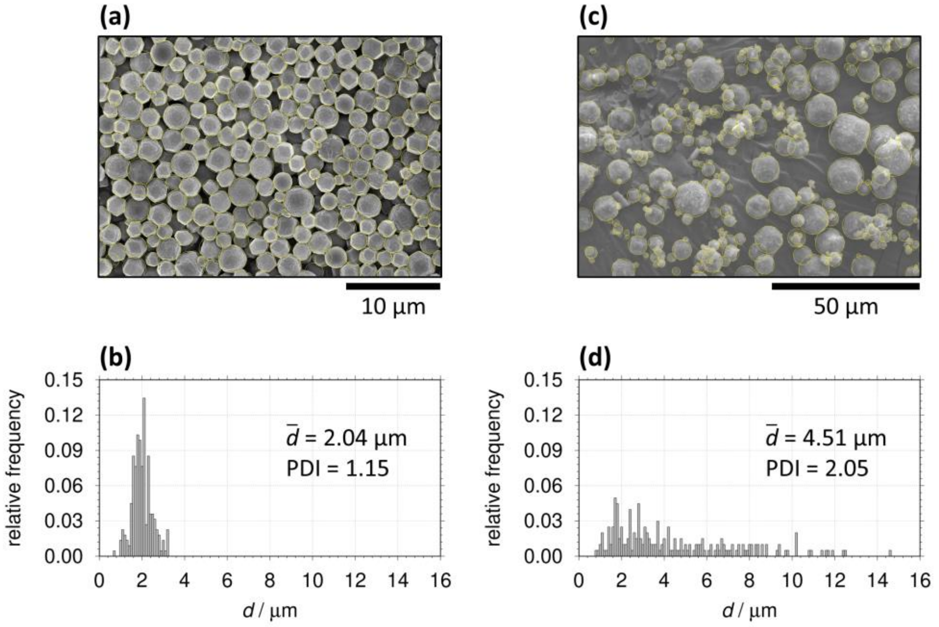

Supplementary Figure S2. Examples of SEM micrographs (a, c) and the corresponding histograms (b, d) of the determined particle size distribution. Less polydisperse example (a, b); experimental conditions: $[Zn^{2+}]_0$ = 2.5 mM, $[2\text{-MeIm}]_0$ = 25 mM, t_{synt} = 24 h, middle zone of the precipitate band. More polydisperse example (c, d); experimental conditions: $[Zn^{2+}]_0$ = 250 mM, $[2\text{-MeIm}]_0$ = 2500 mM, t_{synt} = 24 h, middle zone of the precipitate band. Yellow circles in the SEM micrographs indicate the measured particle diameters, N stands for the number of particles in the indicated diameter (d) range, and N_{tot} is the total particle number.

2 Numerical model and simulations

2.1 Reaction-diffusion (out-of-equilibrium synthesis) of MOFs (partial differential equations)

The out-of-equilibrium synthesis can be described by the following set of partial differential equations (PDEs) in one dimension (in between and perpendicular to the parallel channels):

$$\frac{\partial a}{\partial t} = D_\mathrm{A}\frac{\partial^2 a}{\partial x^2} - k_1 ab\Theta\left(ab - \alpha^*\right) + r_\mathrm{a} \tag{S3}$$

$$\frac{\partial b}{\partial t} = D_\mathrm{B}\frac{\partial^2 b}{\partial x^2} - k_1 ab\Theta\left(ab - \alpha^*\right) + r_\mathrm{b} \tag{S4}$$

$$\frac{\partial c_1}{\partial t} = k_1 ab\Theta\left(ab - \alpha^*\right) + r_1 \tag{S5}$$

$$\frac{\partial c_\mathrm{i}}{\partial t} = r_\mathrm{i},\ \text{if } i \in \{2,\ldots,n\}, \tag{S6}$$

where a, b, and c_i ($\forall\ i \in \{1,\ldots,n\}$) are the concentrations of A, B, and $\mathrm{C_i}$, and the system consists of $n + 2$ (i.e., 102) PDEs. D_A and D_B are the diffusion coefficients of reagents A and B in the gel. Θ is the Heaviside step function, and r_a, r_b, and r_i are the reaction terms describing the concentration change of A, B, and $\mathrm{C_i}$ due to the crystal growth, respectively. α^* is the threshold concentration of the formation of $\mathrm{C_1}$ (the smallest crystal in the model). For the crystal growth processes, we also considered threshold limited reactions with a smaller threshold concentration than α^*. The reaction term (r_i, r_a, and r_b) can be written in a compact form as $\mathbf{r} = kab\Theta(ab - \beta^*)\mathbf{Ac}$, where $\mathbf{c}$ is a concentration vector having its components as the concentrations of the chemical species, $\mathbf{A}$ is the $((n+2)\times n)$ stoichiometric matrix, k stands for the rate coefficients of the crystal growth steps ($k = k_2 = k_3 = \ldots = k_{n-1} = k_n$), and β^* is the threshold concentration of the crystal growth:

$$\mathbf{r} = \begin{pmatrix} r_1 \\ r_2 \\ r_3 \\ \vdots \\ r_{n-2} \\ r_{n-1} \\ r_n \\ r_a \\ r_b \end{pmatrix},\quad \mathbf{c} = \begin{pmatrix} c_1 \\ c_2 \\ c_3 \\ \vdots \\ c_{n-2} \\ c_{n-1} \\ c_n \end{pmatrix},\text{ and }\ \mathbf{A} = \begin{pmatrix} -1 & 0 & 0 & \ldots & 0 & 0 & 0 \\ 1 & -1 & 0 & \ldots & 0 & 0 & 0 \\ 0 & 1 & -1 & \ddots & 0 & 0 & 0 \\ \vdots & \ddots & \ddots & \ddots & \ddots & \vdots & \vdots \\ 0 & 0 & 0 & \ddots & -1 & 0 & 0 \\ 0 & 0 & 0 & \ddots & 1 & -1 & 0 \\ 0 & 0 & 0 & \cdots & 0 & 1 & 0 \\ -1 & -1 & -1 & \cdots & -1 & -1 & 0 \\ -1 & -1 & -1 & \cdots & -1 & -1 & 0 \end{pmatrix}.$$

We solved the set of partial differential equations (Eqs. S3-S6) numerically by using the method of lines technique (Forward Time Centered Space, FTCS) on an equidistant grid with a grid spacing of $\Delta x = 0.01$. We applied the following initial conditions: $a(t = 0, x) = b(t = 0, x) = c_\mathrm{i}(t = 0, x) = 0$ to reflect the initial experimental conditions, namely, there were no chemical species in the gel at $t = 0$. We used Dirichlet boundary conditions for diffusing chemical species at $x = 0$ and $x = L$ as $a(t, x = 0) = b(t, x = L) = a_0$ and $a(t, x = L) = b(t, x = 0) = 0$, where L is the length of the simulation domain. We used the following set of parameters: $D_\mathrm{A} = D_\mathrm{B} = 1$, $\alpha^* = 0.15$, $\beta^* = 0.1$, $k_1 = 10$, $k = k_2 = k_3 = \ldots = k_{n-1} = k_n = 10^2$, $L = 1$, and $\Delta t = 10^{-5}$ (time step).

The diameters of the crystals in the numerical simulations (d_{C_1}, d_{C_2}, …, d_{C_n}) were calculated by assuming that A and B are spheres of unit diameter ($d_\mathrm{A} = d_\mathrm{B} = 1$), and the MOF crystals of increasing sizes ($\mathrm{C_1}$, $\mathrm{C_2}$, …, $\mathrm{C_n}$) are also spheres with volumes defined as $V_{\mathrm{C}_1} = V_\mathrm{A} + V_\mathrm{B} = d_{\mathrm{C}_1}^3\pi/6$, $V_{\mathrm{C}_2} = V_\mathrm{A} + V_\mathrm{B} + V_{\mathrm{C}_1} = d_{\mathrm{C}_2}^3\pi/6$, …, $V_{\mathrm{C}_n} = V_\mathrm{A} + V_\mathrm{B} + V_{\mathrm{C}_{n-1}} = d_{\mathrm{C}_n}^3\pi/6$. With this, the formula for the diameter of the ith crystal can be given as $d_\mathrm{i} = \sqrt[3]{2i}$. The average particle size ($\bar{d}$) and the polydispersity index (PDI) were calculated similarly as in case of the experiments, see Eqs. (S1) and (S2). The particle size is in dimensionless unit.

2.2 Bulk synthesis of MOFs (ordinary differential equations)

To simulate the formation of MOFs in the bulk (homogeneously well-mixed) system, we used a set of ordinary differential equations (ODEs), we modified Eqs. (S3) and (S5) by removing the diffusion term from them:

$$\frac{\mathrm{d}a}{\mathrm{d}t} = -\ k_1 ab\Theta\left(ab - \alpha^*\right) + r_\mathrm{a} \tag{S7}$$

$$\frac{\mathrm{d}b}{\mathrm{d}t} = -\ k_1 ab\Theta\left(ab - \alpha^*\right) + r_\mathrm{b} \tag{S8}$$

$$\frac{\mathrm{d}c_1}{\mathrm{d}t} = k_1 ab\Theta\left(ab - \alpha^*\right) + r_1 \tag{S9}$$

$$\frac{\mathrm{d}c_\mathrm{i}}{\mathrm{d}t} = r_\mathrm{i},\ \text{if } i \in \{2,\ldots,n\}. \tag{S10}$$

We solved the ODEs (Eqs. S7-S10) numerically by using the CVODE solver ($atol = 10^{-10}$ and $rtol = 10^{-8}$). We applied the following initial conditions $a(t = 0) = b(t = 0) = a_0$, and $c_\mathrm{i}(t = 0) = 0$ to reflect the initial experimental conditions in the bulk synthesis, namely, there were only the reagents in the system at $t = 0$. The time step was automatically adjusted by the solver. The diameters of the crystals, the average particle size ($\bar{d}$), and the polydispersity index (PDI) were calculated as described in Section 2.1 in the SI. The particle size is in dimensionless unit.

3 Supplementary Figures

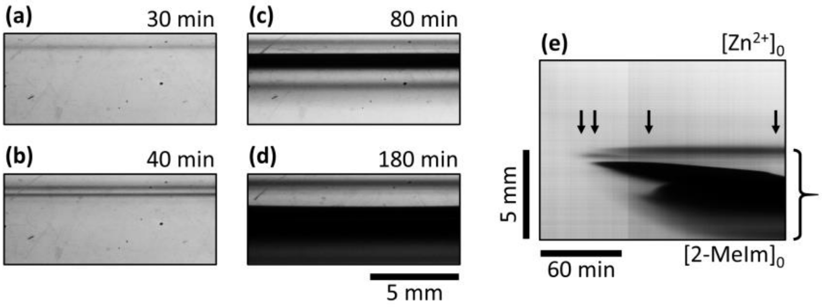

Supplementary Figure S3. Moving precipitate fronts and periodic precipitation of ZIF-8 in the two-channel open gel reactor. The 2 m/V% agarose gel and the reagent solutions were made by using deionized water. The boundary concentrations were $[Zn^{2+}]_0$ = $[2\text{-MeIm}]_0$ = 1 M, the synthesis time was 180 min. Snapshots of the bottom half of the inter-channel zone made at the indicated times (a-d) and space-time plot of the whole inter-channel zone about the whole experiment (e). The white precipitate appears as black in the pictures because transmitted light was monitored. The positions of the channels are indicated by $[Zn^{2+}]_0$ and $[2\text{-MeIm}]_0$ labels. In the space-time plot, the bracket and arrows show the spatial domain and times of the snapshots.

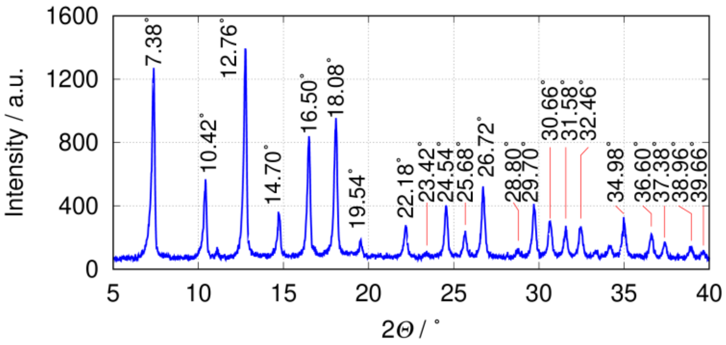

Supplementary Figure S4. PXRD pattern of the out-of-equilibrium synthesized ZIF-8 crystals. The boundary concentrations were $[Zn^{2+}]_0$ = 25 mM and $[2\text{-MeIm}]_0$ = 250 mM, the synthesis time was t_{synt} = 24 h. The 2Θ values of the characteristic peaks are written in the figure.

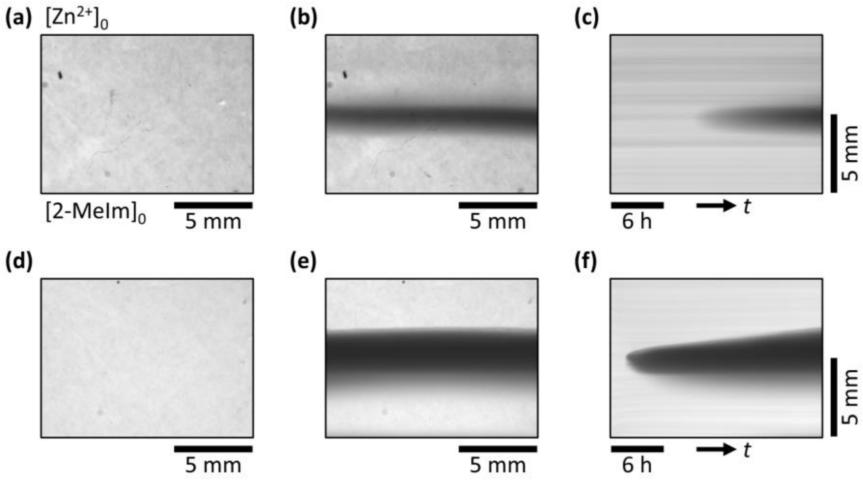

Supplementary Figure S5. Macroscopic characteristics of the forming precipitate band in the experiments, depending on the boundary concentrations and time: at $[Zn^{2+}]_0$ = 2.5 mM (a-c) and $[Zn^{2+}]_0$ = 500 mM (d-f). Snapshots at the beginning (t = 0, empty gel) (a, d) and at the end (t_{synt} = 24 h) (b, e) of each experiment. Space-time plots of the temporal evolution in each case (c, f). The experiments were made in DMF:H_2O (1:1) solvent (see Section 1.1 in the SI). The ratio of the boundary concentrations was fixed to $[Zn^{2+}]_0$:$[2\text{-MeIm}]_0$ = 1:10. The white precipitate appears as black in the pictures because transmitted light was monitored. The positions of the channels are indicated by $[Zn^{2+}]_0$ and $[2\text{-MeIm}]_0$ labels.

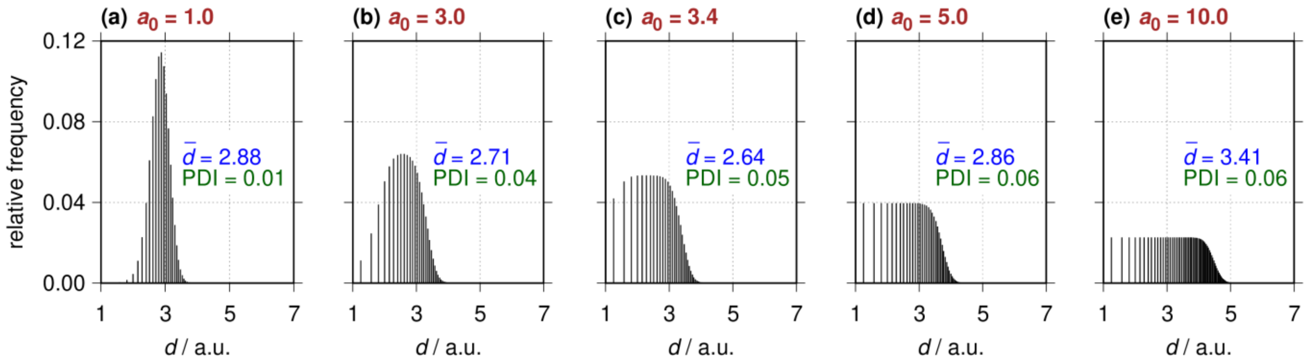

Supplementary Figure S6. Numerical simulations of the particle size distribution as the function of the boundary concentration in the RD model. The histograms (a-e) show the size distribution at x = 0.5, which is the middle of the domain. The boundary concentrations were a_0 for both reactants. The applied concentrations (a_0), the calculated average particle size ($\bar{d}$), and the polydispersity index (PDI) are given in each figure. The particle size is in dimensionless unit.

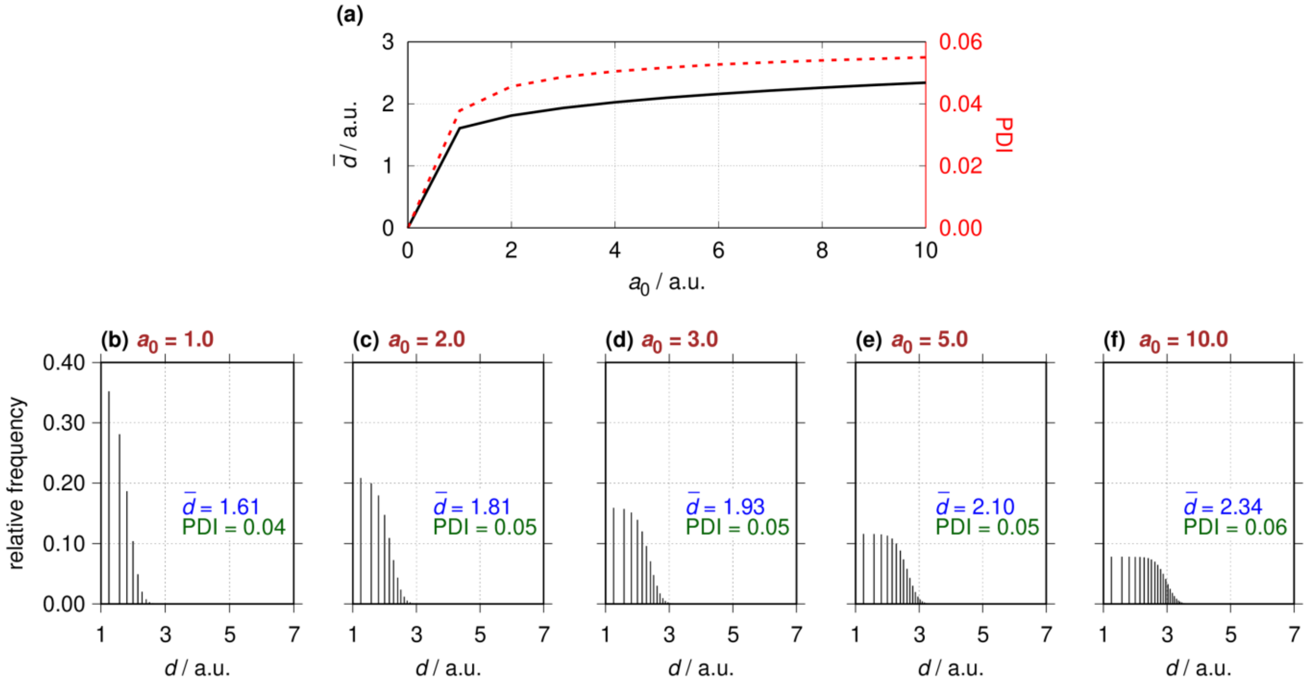

Supplementary Figure S7. Numerical simulations of the particle size distribution as the function of the initial concentration in the bulk model. The initial concentrations were a_0 for both reactants. The concentration dependence of the average particle size and the polydispersity (a). Histograms of the particle size distribution at some representative a_0 values (b-f). The calculated values of the average particle size ($\bar{d}$) and the polydispersity index (PDI) are given in each figure (b-f). The particle size is in dimensionless unit.

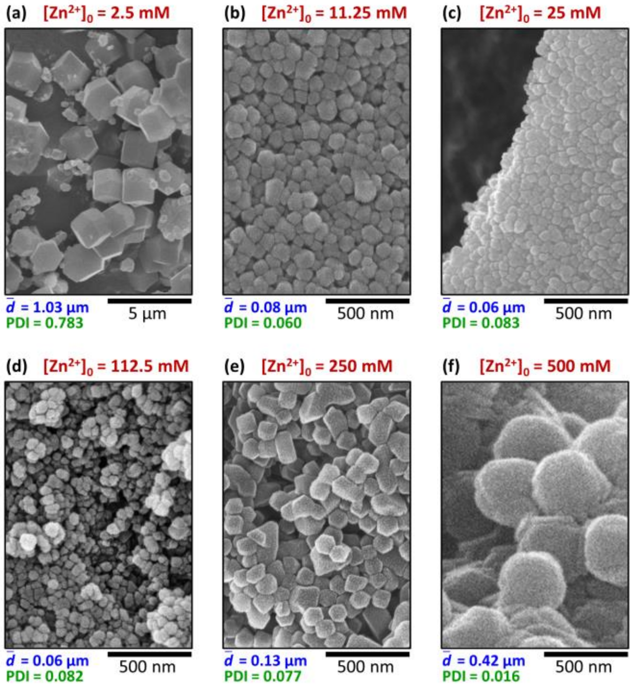

Supplementary Figure S8. Concentration dependence of the particle size in the well-mixed bulk synthesis represented in SEM micrographs (a-f). The ratio of concentrations was fixed to $[Zn^{2+}]_0$:[2-MeIm]$_0$ = 1:10, and the synthesis time was 24 h. The average particle size ($\bar{d}$) and the polydispersity index (PDI) are given below the SEM micrographs.

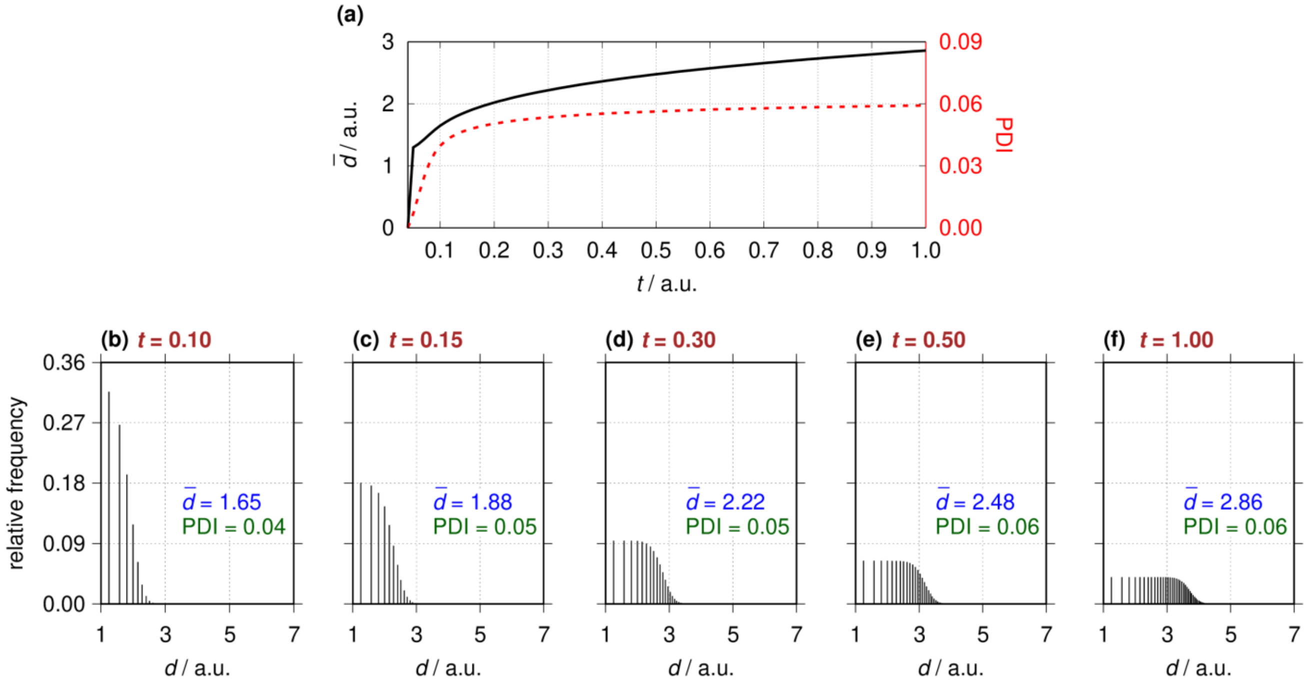

Supplementary Figure S9. Numerical simulations of the particle size distribution as the function of time in the RD model. The temporal evolution of the average particle size and the polydispersity at x = 0.5 position (a). The boundary concentrations were a_0 = 5.0 for both reactants. Histograms of the particle size distribution at some representative t values (b-f). The calculated values of the average particle size ($\bar{d}$) and the polydispersity index (PDI) are given in each figure (b-f). The particle size is in dimensionless unit.

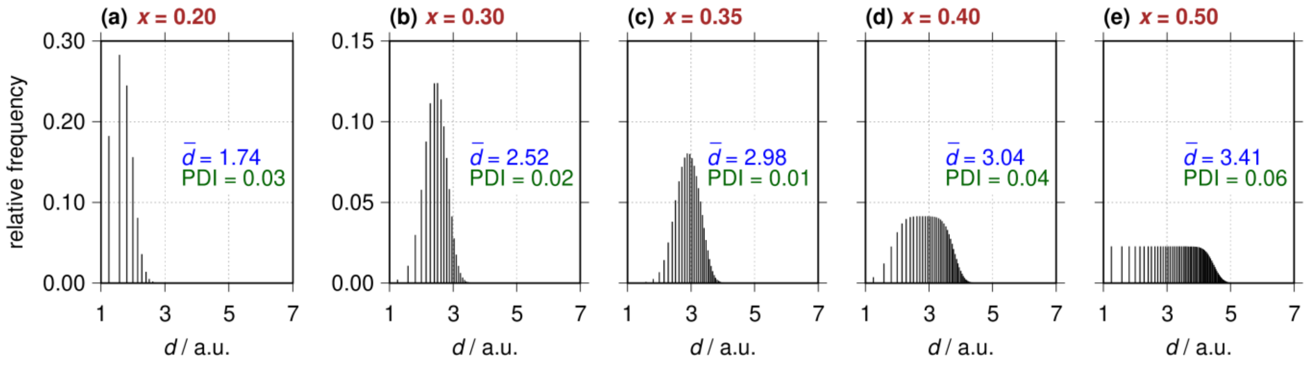

Supplementary Figure S10. Numerical simulations of the particle size distribution as the function of the spatial position along the direction of the cross-gradients (x) in the RD model. The boundary concentrations were a_0 = 10.0 for both reactants. A and B are commutable in the model, so our results are symmetric with respect to x = 0.5, that is why we show only the $0 < x \leq 0.5$ regime. The spatial position (x), the calculated average particle size ($\bar{d}$), and the polydispersity index (PDI) are given in each figure. The particle size is in dimensionless unit.

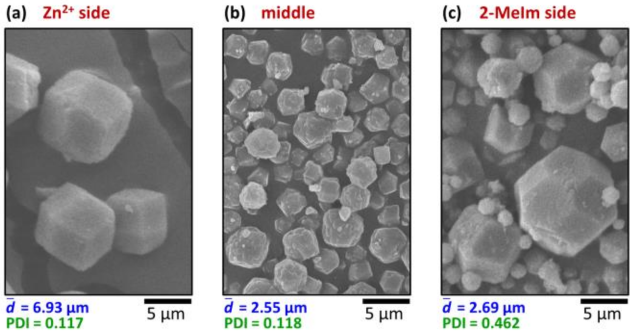

Supplementary Figure S11. Particle size at different spatial positions along the direction of the cross-gradients (Zn^{2+} side, middle zone, and 2-MeIm side) in the out-of-equilibrium synthesis represented in SEM micrographs (a-c). The boundary concentrations were fixed to $[Zn^{2+}]_0$ = 2.5 mM and $[\text{2-MeIm}]_0$ = 25 mM, and the synthesis time was 168 h. The average particle size ($\bar{d}$) and the polydispersity index (PDI) are given below the SEM micrographs.

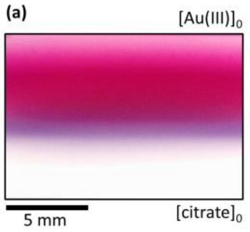

Supplementary Figure S12. Controlled gold nanoparticle synthesis in the two-channel gel reactor in the agarose gel. Top view of the inter-channel zone at the end of the experiment that was 6 h. The pictures were recorded with a color camera. The labels $[\text{Au(III)}]_0$ and $[\text{citrate}]_0$ indicate the positions of the channels. The boundary concentrations were $[\text{Au(III)}]_0$ = 1 mM and $[\text{citrate}]_0$ = 4 mM, respectively.

4 References

1 Raval, N. *et al.* in *Basic Fundamentals of Drug Delivery* (ed Rakesh K. Tekade) 369-400 (Academic Press, 2019).

2 Kesse, X., Vichery, C. & Nedelec, J.-M. Deeper Insights into a Bioactive Glass Nanoparticle Synthesis Protocol To Control Its Morphology, Dispersibility, and Composition. *ACS Omega* **4**, 5768-5775, doi:10.1021/acsomega.8b03598 (2019).
